# The potential regulatory role of RNA methylation in ovarian cancer

**DOI:** 10.1080/15476286.2023.2213915

**Published:** 2023-05-16

**Authors:** Shijie Zhao, Mengxue Zhang, Xiaolan Zhu, Jie Xing, Jiaming Zhou, Xinming Yin

**Affiliations:** a Department of Central Laboratory, The Fourth Affiliated Hospital of Jiangsu University, Zhenjiang, Jiangsu, China; b Reproductive Medicine Center, The Fourth Affiliated Hospital of Jiangsu University, Zhenjiang, Jiangsu, China; c Department of Gynaecology, The Fourth Affiliated Hospital of Jiangsu University, Zhenjiang, Jiangsu, China

**Keywords:** RNA methylation, ovarian cancer, M6a, M5c, M7g, M1a

## Abstract

Updates in whole genome sequencing technologies have revealed various RNA modifications in cancer, among which RNA methylation is a frequent posttranscriptional modification. RNA methylation is essential for regulating biological processes such as RNA transcription, splicing, structure, stability, and translation. Its dysfunction is strongly associated with the development of human malignancies. Research advances with respect to the regulatory role of RNA modifications in ovarian cancer include N6-methyladenosine (m6A), 5-methylcytosine (m5C), N1-methyladenosine (m1A), and N7-methylguanosine (m7G). Numerous studies have demonstrated that epigenetic modifications of RNA can influence the progression and metastasis of ovarian cancer and may provide excellent targets for cancer therapy. This review highlights advances in research on RNA methylation modifications and ovarian cancer prognosis, carcinogenesis, and resistance, which could provide a theoretical foundation for designing therapeutic strategies for ovarian cancer based on RNA methylation modifications.

## Introduction

One of the three main gynaecologic malignancies, ovarian cancer (OC), is also the most common cause of death from gynaecologic tumours [1]. According to statistics, there will be approximately 53,342 new cases and approximately 37,519 deaths from OC in China in 2020, accounting for 17.63% and 18.10% of the global cases respectively, and the cancer burden will increase annually [2]. However, prognosis is dismal because ovarian cancer is usually advanced when diagnosed due to the lack of early diagnostic methods. Despite improvements in surgery, chemotherapy, novel immunotherapies, and overall survival at all stages, various molecular processes in ovarian cancer that contribute to tumour growth and therapeutic targets are still unknown [3,4].

DNA, RNA and protein postsynthetic chemical modifications are involved in alterations at three levels: transcriptional, posttranscriptional and posttranslational, respectively. Of these, posttranscriptional regulation involves RNA modifications and ncRNA, and this mechanism of translational control is known as epitranscriptomics. Over the past five decades, more than 100 RNA postsynthetic modifications have been identified in different RNA classes [5]. All kinds of known RNA have been discovered to be modified, with the vast majority of modifications occurring only in ribosomal RNA (rRNA) and/or transfer RNA (tRNA). Recent studies suggest that RNA modifications may affect molecular processes such as RNA metabolism, splicing, stability, and translation [6,7]. Methylation modifications are among the most abundant RNA modifications. The most common internal modifications in eukaryotic RNAs include N6-methyladenosine (m6A), 5-methylcytosine (m5C), 7-methylguanosine (m7G), N1-methyladenosine (m1A) and others [8]. mRNA modifications, especially m6A modifications, are the most prevalent internal modifications in mRNAs. The primary roles of m6A are to regulate DNA damage, proliferation, cell survival, migration and invasion, progression, and chemoresistance in cancer [9].

The dynamic modification process of RNA necessitates the involvement of numerous particular proteins known as RNA modifying proteins (RMPs). Among these, methyltransferases (also known as writers), demethylases (commonly known as erasers), and binding proteins (usually known as readers) are involved in the insertion, deletion, and recognition of RNA modifications, respectively [7,10]. The advent of analytical methods such as MeRIP-seq, miCLIP, and other specific sequencing allows for the study of the location and quality of methylation modifications on RNA, as well as the identification of writers, erasers, and readers that modulate the transcriptomic mark [11,12]. RNA methylation modifications and their associated regulatory proteins are aberrantly expressed in cancer and are crucial to the development of cancer [13,14]. According to the RNA-seq data used by GEPIA (https://gepia.cancer-pku.cn), the RNA modification proteins YTHDC1/2, HNRNPCA2B1, IGF2BP2/3 and YBX1 were significantly highly expressed and only METTL3/16 expressed at low levels in ovarian cancer tissues; other regulatory enzymes were not significantly differentially expressed. Interestingly, these regulators show inconsistent expression trends and RNA metabolic effects in other tumours. For instance, catalytic m6A-modified methyltransferase-like 3 (METTL3) is highly expressed in malignancies such as cholangiocarcinoma, thymoma, and diffuse large B-cell tumours according to the GEPIA website (https://gepia.cancer-pku.cn). Previous evidence suggests that METTL3 mediates IFIT2 mRNA decay in an m6A-dependent manner to inhibit the progression of intrahepatic cholangiocarcinoma [15]. Aly/REF Export Factor (ALYREF) promotes mRNA export by acting as a reader of m5C and is associated with prognosis in hepatocellular and glial carcinomas [16–18]. Similarly, promoter hypermethylation of the m1A demethylase ALKBH3 is an independent factor associated with poor prognosis in Hodgkin’s lymphoma [19]. METTL1 and WDR4 are complex catalyst of m1A modification, and METTL1 overexpression in bladder cancer is linked to a poor prognosis and mediates m7G tRNA modification [20].

Numerous investigations have confirmed that RNA methylation modifications, such as m6A, m5C, m1A, and m7G, are related to various cancers and are involved in their biological processes [13]. However, research on the role of RNA methylation in the progression of ovarian carcinogenesis remains incomplete. In this review, we will discuss the cellular functions and molecular mechanisms of RNA modifications in regulating gene expression in ovarian cancer. Our review will focus on m6A, m5C, m1A, and m7G modified coding and noncoding RNAs, with emphasis on the molecular processes of these RMPs in cell proliferation, migration and invasion, stem cell differentiation, immunity, and drug resistance of OC. In addition, RNA-modified regulators will also be of interest as potential molecular targets and therapeutic targets for ovarian cancer will also be of interest.

## RNA methylation in ovarian cancer

### N6-methyladenosine methyltransferase

The methyltransferase complex (MTC), which has METTL3 as its core methyltransferase subunit, is responsible for installation of m6A on mRNA. Regulators such as METTL14/16, Wilms tumor1 associated protein (WTAP), VIRMA(KIAA1429), RNA binding motif protein 15(RBM15), and zinc finger CCCHType Containing 13 (ZC3H13) perform different biological functions during m6A modification. The regulator METTL3 is responsible for catalysing m6A modification [21]. METTL3 demonstrates hypomethylation and elevated expression in the tissues and cells of ovarian cancer [22]. METTL3 has been shown in various studies to contribute to the tumorigenesis of ovarian cancer. In addition, METTL3 mediates m6A modification in endometrioid epithelial ovarian cancer(EEOC) without the assistance of WTAP or METTL14 [23]. METTL14 can assist METTL3 in substrate recognition and expressed at low levels in ovarian cancer [24,25]. New studies have shown that METTL16 catalyzes m6A on mRNAs in the nucleus and encourages mRNA translation in the cytoplasm [26]. The METTL3-METTL14 heterodimer is promoted by WTAP to the nuclear speckle [27]. VIRMA directs methyltransferase components to specific RNA regions [24]. WTAP and VIRMA are substantially expressed in ovarian cancer and correlate with prognosis [28,29]. RBM15 recruits the complex to specific RNA locations by binding the m6A complex, and its increased expression is associated with ovarian cancer metastasis [30,31]. ZC3H13 anchors the complex containing WTAP in the nucleus to promote m6A modification [32]. In ovarian cancer, the expression of ZC3H13 was found to correspond with poor overall survival of patients and the degree of immune infiltration [31,33].

### N6-methyladenosine demethylase

Fat mass and obesity-associated protein (FTO), as the first discovered ‘eraser’, was found to delete the m6A modification by using the nuclear RNA form of m6A as a substrate [34]. In addition, ALKBH5, another demethylase in the ALKB family, was found to significantly affect mRNA export and RNA metabolism in mammals [35]. Patients with ovarian cancer had low levels of FTO and ALKBH5, whereas those with higher levels of both presented shorter overall survival and progression-free survival (PFS) [36]. In various ovarian cancer studies, the erasers FTO and ALKBH5 regulate various biological processes.

### N6-methyladenosine reader

Identified ‘readers’ that recognize m6A modification sites include the YTHDF1/2/3 family of YT521-B homologous (YTH) structural domain proteins, the YTH structural domain protein YTHDC1/2, the insulin-like growth factor 2 mRNA binding protein IGF2BP1/2/3 and the heterogeneous nuclear ribonucleoprotein (HNRNP) family. While YTHDF1 enhances mRNA translation and YTHDF2 reduces mRNA stability, YTHDF3 synergizes with YTHDF1 to facilitate protein synthesis and affects YTHDF2-mediated mRNA decay [37,38]. YTHDF1 and YTHDF2 are significantly expressed in ovarian cancer and may be associated with prognosis, while YTHDF3 may increase the pathological staging of ovaries [39,40]. YTHDC1 promotes RNA splicing and translocation, and YTHDC2 enhances the translation of target RNAs [41,42]. YTHDC1 promotes pre-RNA splicing and export; YTHDC2 enhances context-dependent mRNA translation or recognizes partial mRNAs to accelerate their degradation [41,42]. IGF2BP1/2/3 recognizes m6A sites through specific KH structural domains and enhances mRNA stability and translation [43]. IGF2BP1 enhances OC cell invasion by antagonizing miRNA damage gene expression [44]. The HNRNP family contains HNRNPC, HNRNPG and HNRNPA2B1, which modulate pre-mRNA processing through the ‘m6A switch mechanism’ [45,46]. The ability of HNRNPA2B1 to bind to the vicinity of m6A sites is enhanced to facilitate nuclear events such as primary microRNA processing [47]. HNRNPA2B1 promotes the OC malignant phenotype through upregulation of Lin28B expression [48]. In addition, HNRNPC is considered a predictor of paclitaxel resistance [31].

### 5-methylcytosine

5-Methylcytosine (m5C), the methylation of cytidine at position 5, is a posttranscriptional modification. The NOP/SUN RNA methyltransferase (NSUN) family, which includes members NSUN1 through NSUN7, DNA methyltransferase 2 (DNMT2), and tRNA aspartic acid methyltransferase 1, is responsible for catalysing the m5C modification [49]. NSUN2, a major mRNA methyltransferase, is associated with stress response pathways and stem cell differentiation, and it may impact the growth, maintenance, and progression of tumours [50]. DNMT3A/3B interacts with microRNA-29b in a double negative feedback manner, leading to OC progression [51]. m5C-modified binding proteins (YTHDF2, ALYREF, YBX1) participate in m5C modification in a methylase-dependent or independent manner. According to reports, ALYREF is a specific mRNA m5C binding protein [16]. Y-box binding protein 1(YBX1) maintains RNA stability by recruiting the ELAVL1 protein to recognize m5C modifications. YBX1 nuclear translocation is regulated by Akt activation and affects the expression of drug resistance genes in OC cells [52]. In ovarian cancer, the potential role of m5C regulators may influence metabolic heterogeneity and drug chemotherapy sensitivity and correlate with prognosis in OC patients [53].

### N1-methyladenosine

N1-methyladenosine (m1A) is the methylation of the nitrogen atom at position 1 of adenosine on tRNA. Transfer RNA methyltransferase 10 (TRMT10) and the transfer RNA methyltransferase 6/61 (TRM6/61) complex are currently known methyltransferases. Different studies have shown that m1A is mainly present in the 5’-UTR structural domain associated with high levels of translation, while other m1A sites are mostly concentrated in the CDS and 3’-UTR regions [54,55]. m1A modifications on tRNAs can be deleted by ALKBH1/3 [56,57]. In ovarian cancer, ALKBH3-mediated m1A demethylation enhances CSF-1 mRNA stability [58]. In addition, studies have confirmed that YTHDF2 can recognize specific methylation by m1A and affect transcript stability [59].

### 7-methylguanosine

**Figure 1.**
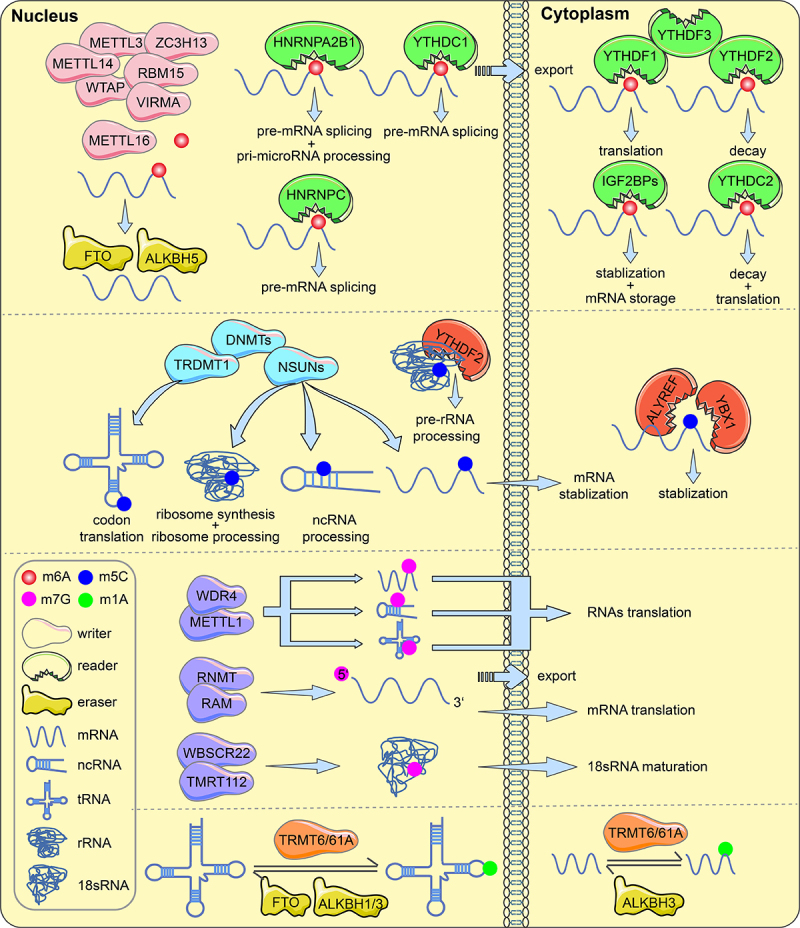
RNA modifications with functions of regulators (writers, erasers and readers). m6A, m5C, m7G and m1A methylations in mRNA, tRNA, rRNA and ncRNA are involved in RNA processing and metabolism.

The majority of 7-methylguanosine (m7G) is located at the 5’ terminal structure (also called the “cap”) of mRNA, and the “cap” reduces exonuclease degradation to improve mRNA stability [60–62]. WDR4, RNMT, RAM, WBSCR22, and TMRT112 have been identified as m7G methyltransferases [63,64]. Global translation is ultimately controlled by the METTL1/WDR4 complex, which adds m7G modifications to mRNA (internal site), tRNA (G46 site), and miRNA (G-quadruplex structure). The RNMT/RAM complex regulates m7G modifications of the 5’ cap of mRNA, which mediates the process of mRNA nuclear export and translation. The WBSCR22/TMRT112 complex adds m7G modifications at the G1638 site of 18S rRNA to promote 18S rRNA maturation [65]. These m7G regulators play different roles in various other cancers. Figure 1 show the roles of m6A, m5C, m1A and m7G modified proteins in RNA metabolism.

## Relationship between RNA methylation modifications and the prognosis of ovarian cancer

To explore the clinicopathological characteristics and prognostic values of methylation regulators in ovarian cancer, the cBioPortal tool was used to evaluate RNA-seq and clinicopathological data from TCGA, consensus clustering to distinguish expression, regression models to determine prognostic relevance, ROC curves, and risk scores to predict survival, and the TIMER online database and nomogram were used to integrate data. These analyses suggest that RNA m6A methylation regulators are associated with clinicopathology and prognosis in ovarian cancer [29]. A meta-analysis showed that aberrant METTL3 expression in different cancers, including ovarian cancer, tended to predict poor prognosis [66]. Comprehensive biological information analysis and gene enrichment analysis (GSEA) showed that high WTAP expression was significantly associated with poor outcomes in ovarian cancer and cell cycle regulation [67]. According to the study, IGF2BP2, KIAA1429, and YTHDF1 regulators are highly amplified in OC, and the majority of m6A genes, including METTL3, KIAA1429, HNRNPC, ZC3H13, and IGF2BP2, are upregulated in ovarian cancer tissues. These m6A-modified proteins could be clinical prognostic factors in ovarian cancer [68]. In OC, the m6A regulators IGF2BP1, VIRMA, and ZC3H13 were connected to prognosis and the tumour-associated WNT pathway [69]. More studies have shown that high expression levels of WTAP, FTO, ALKBH5, and YTHDF3 are related to shorter OS and PFS, and the m6A action of RBM15B, ZC3H13, YTHDF1, and IGF2BP1 may mediate immune infiltration [31]. METTL14 was highly expressed in samples from individuals with EOC and was associated with a poorer prognosis [70]. Microarray data analysis revealed that METTL14 was less enriched in OC tissues than in normal tissues, and its low expression reduced overall patient survival [25]. According to risk score models, patients with ovarian cancer who had low VIRMA or high HNRNPA2B1 expression had prolonged half survival, and the constructed miRNA-m6A regulator (VIRMA, IGF2BP1, HNRNPA2B1)-target gene regulatory network could predict the prognosis of OC patients [71]. In addition, noncoding RNA lncRNAs play an essential role in the development of various cancers, and m6A is the most widely modified modality. Yang et al. investigated the potential mechanisms involved in m6A modification of lncRNAs in OC and identified seven m6A-associated lncRNA gene signatures that could predict the prognosis of OC patients [72,73]. In the most recent study, an RNA modification pattern scoring system (called the RMW score) based on 26 RNA modification ‘writers’ of m6A, m1A, APA, and A-I was created to measure individual OC patients, which correlated with the tumour immune microenvironment (TME), predicted drug sensitivity in OC patients, and predicted survival in combination with prognostic clinical features, and as an individualized treatment strategy [74]. Compared to normal tissues, epithelial ovarian cancer exhibits a high level of m5C expression, and its expression is correlated with pathological stage, tumour grade, and lymph node metastasis [75]. M5C is involved in ovarian cancer progression and correlates with tumour prognosis; among the four m5C regulators, the FC fragment of IgG-binding protein FCGBP and HOXB3 are risk factors for the prognostic risk score, while TYMSOS and CLDN19 are protective factors for the prognostic risk score [76]. Nuclear YBX1 expression level may be an independent factor for poor prognosis of OC patients [77]. The lncRNA COL4A3, which is regulated by m5C modification and enriched in multiple pathways, has a clinically significant relationship with the prognosis of HGSOC [78]. Analyses in public databases and cellular functional characterization verified that overexpression of the m1A-modified reader TRMT10C predicted poorer survival in gynaecologic cancers [79]. The m1A regulators ALKBH1/3, TRMT6, TRMT10C, and YTHDF1/2 are pro-oncogenes, and OC patients with high expression of these regulators have a poor prognosis [80]. In ovarian plasmacytic cystic adenocarcinoma, a substantial correlation was observed between the expression of the m7G modifier METTL1 and OS and disease-specific survival (DSS) [81]. Table 1 show the main role of regulators of RNA modification related with prognosis of ovarian cancer. In summary, various RNA methylation regulators can predict survival in ovarian cancer patients, which provides a basis for understanding tumour progression, aiding diagnosis, and developing treatment plans.

**Table 1.** The main role of regulators of RNA modification related with prognosis of ovarian cancer.

Modification Type	Regulator	Type of regulator	Role in survival	Role in tumour	Expression in cancer	Ref
m6A	METTL3	Writer	Poor	Oncogene	Upregulated	[65,67]
	METTL14	Writer	Poor	Oncogene	Upregulated Downregulated	[24,69]
	WTAP	Writer	Poor	Oncogene	Upregulated	[66]
	RMB15	Writer	Poor	Oncogene	Upregulated	[30]
	ZC3H13	Writer	Poor	Oncogene	Upregulated	[67]
	KIAA1429/ VIRMA	Writer	Poor	Oncogene	Upregulated Downregulated	[25,67]
	FTO	Eraser	Poor	Oncogene	Upregulated	[30]
	ALKBH5	Eraser	Poor	Oncogene	Upregulated	[30,82]
	YTHDF1/3	Reader	Poor	Oncogene	Upregulated	[30,67]
	IGF2BP1/2	Reader	Poor	Oncogene	Upregulated	[67,68]
	HNPNPA2B1	Reader	Poor	Oncogene	Upregulated	[25]
m5C	NSUN2	Reader	-	-	Upregulated	[83]
	YBX1	Reader	Poor	Oncogene	-	[84]
m1A	TRMT6	Writer	Poor	Oncogene	Upregulated	[79]
	TRMT10C	Writer	Poor	Oncogene	Upregulated	[78,79]
	ALKBH1/3	Eraser	Poor	Oncogene	Upregulated	[79]
	YTHDF1/2	Reader	Poor	Oncogene	Upregulated	[79]
m7G	METTL1	Writer	Poor	-	Upregulated	[80]

## Role of RNA methylation modifications in ovarian cancer carcinogenesis

It is generally known that m6A plays a crucial role in tumours and display different characteristics in different cancers. In the last decade, m5C modifications have been linked to a variety of cancer types, but the precise mechanisms are still unclear. Although the study of m7G and m1A in cancer is not deep and comprehensive, the critical role of m1A and m7G in the biological processes of tumour cannot be ignored [13,65]. A growing number of studies have shown correlations between RNA methylation and cancer development and progression. The following sections will describe in detail the roles of various RNA methylation regulators in OCs and their effects.

### Role of RNA methylation modifications in ovarian cancer proliferation, invasion and migration, and apoptosis

**Figure 2.**
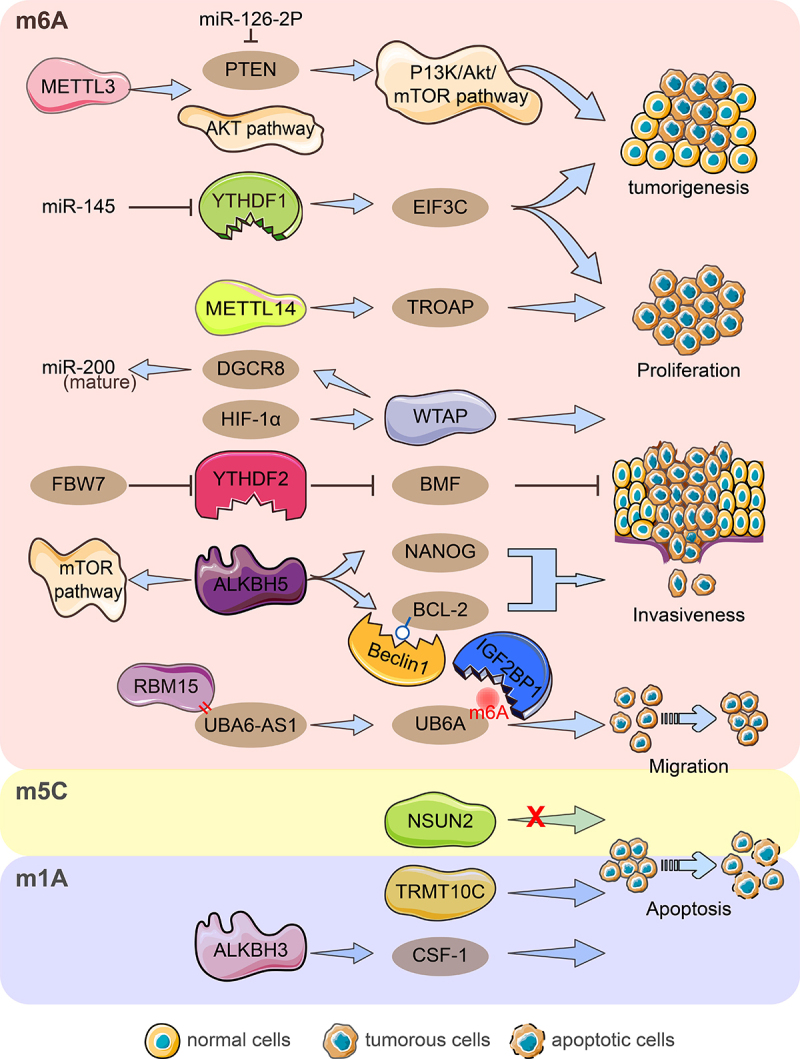
Diagram of RNA modification regulators important for ovarian cancer growth, invasion, migration, and apoptosis. By hindering the miR-126-5p-targeted suppression of PTEN and hence obstructing the PI3K/Akt/mTOR pathway, METTL3 aids in the development and carcinogenesis of ovarian cancer. The AKT pathway was activated by METTL3, which also encouraged proliferation, invasion, and migration. By focusing on TROAP, METTL14 encouraged procreation, invasion, and migration while hindering procreation capacity. HIF-1‘s upregulation of WTAP expression interferes with miRNA processing and speeds up tumour growth, invasion, and migration. By enlisting RBM15, UBA6-AS1 boosted the m6A methylation of UBA6 mRNA. IGF2BP1 was discovered to be the m6A reader protein of the UBA6-AS1-RBM15-mediated m6A modification of UBA6 mRNA, which improved UBA6 mRNA stability and inhibited the proliferation, migration, and invasion of OC cells functionally. YTHDF1 aided in the carcinogenesis of ovarian cancer by promoting the proliferation, invasion, and migration of cells, as well as increasing the translation of EIF3C in an m6A-dependent manner. MiR-145 inhibited YTHDF2‘s ability to increase the proliferation and migration of EOC cell lines, and FBW7 inhibits YTHDF2-mediated BMF mRNA degradation in ovarian cancer to slow tumour growth and development. Through raising NANOG mRNA expression, ALKBH5 encouraged OC cells to be aggressive and anti-apoptosis. The expression of ALKBH5 was increased, which facilitated the growth and invasion of epithelial ovarian cancer cells. ALKBH5 also activates the mTOR pathway to control autophagy and facilitates the connection between Bcl-2 and Beclin1, which is dependent on m6A. In ovarian cancer, NSUN2 did not significantly control cell migration and proliferation. TRMT10C encouraged OC migration, invasion, and proliferation. In order to encourage the invasiveness of ovarian cancer cells, ALKBH3 improved the stability of CSF-1.

Methyltransferase-like 3 (METTL3) mediates the maturation and upregulation of miR-126-2p to activate the PI3K/Akt/mTOR pathway by directly binding to phosphatase and tensin homologues (PTEN) to promote ovarian cancer progression and tumorigenesis; moreover, knockdown of METTL3 inhibits ovarian tumour formation *in vivo* [22]. Downregulation of METTL3 inhibited cell proliferation, colony formation, and invasion, induced apoptosis, and suppressed AKT pathway activation in ovarian cancer [85]. In the EOC cell lines A2780 and SKOV3, high expression of METTL14 promoted cell proliferation invasion and migration [70]. METTL14 targeted TROAP mRNA through m6A modification to reduce stability, thus inhibiting cell proliferation and suppressing the proliferative capacity of ovarian cancer cells *in vitro* and *in vivo* [25]. Interestingly, METTL14 and WTAP expression did not differ significantly in EEOC and adjacent tissues [23]. Hypoxia-inducible factor (HIF)-1α activates WTAP transcription causing enhanced proliferation and migration of ovarian cancer in *vitro*, and WTAP targets DGCR8 in an m6A-modified manner to promote maturation of precursor miRNAs [86]. LncRNA ubiquitination modifier activator 6 antisense RNA1 (UBA6-AS1) recruits RBM15 to mediate UBA6 m6A methylation, and IGF2BP1 enhances UBA6 stability by recognizing its m6A modification, ultimately encouraging ovarian cancer cell proliferation, invasion, and migration [87]. Coculture of OC cells with M2 macrophages enhanced the proproliferative and antiapoptotic ability of ALKBH5 and promoted tumour growth *in vivo*. Increased NANOG RNA expression by ALKBH5 demethylation leads to enhanced aggressiveness of OC cells [82]. ALKBH5 enhances BCL-2 mRNA stability in an m6A-demethylation manner and promotes autophagy-inhibiting complex Beclin1-BCL-2 interactions. High ALKBH5 level expression in OC tissues mediates BCL-2 upregulation, which regulates OC proliferation, invasion and autophagy, and controls the mTOR pathway involved in autophagy [88]. Knockdown of YTHDF1 reduced OC cell, invasive and migratory abilities *in vitro*, and inhibited tumorigenesis and metastasis *in vivo*. YTHDF1 recognizes the target mRNA EIF3C in an m6A-dependent manner to facilitate its protein synthesis and plays an essential role in ovarian cancer growth and migration [39]. YTHDF2 significantly downregulated total mRNA m6A levels to enhance EOC cell proliferation and migration, and its overexpression rescued the tumour suppressive effect induced by the target gene miR-145 [89]. Recent research reports that the tumour suppressor FBW7 mediates YTHDF2 protein hydrolysis via ubiquitination to inhibit ovarian cancer proliferation and growth; In contrast, FBW7 inhibits BMF mRNA degradation in an m6A-YTHDF2 manner to support ovarian cancer apoptosis, which is a downstream target of YTHDF2 [40]. Codifferentially expressed genes with overlapping m5C peaks and RNA-seq in plasmacytoid ovarian cancer correlated with cell growth, maintenance, cytoplasmic membrane, and cell adhesion molecule activity [83]. The results in the GEO and TCGA databases showed that NSUN2 was significantly upregulated in ovarian cancer, and its protein levels were positively correlated with cancer stage. At the same time, there was no effective regulation of cell proliferation and migration in ovarian cancer [90]. Knockdown of the m1A writer TRMT10C inhibited proliferation, colony formation, and migration of ovarian and cervical cancer cells [79]. Moreover, the m1A ‘eraser’ ALKBH3 enhanced the stability of cytokine CSF-1 and the invasive ability of cancer cells but it had no effect on cell proliferation and migration [58] (Figure 2). Obviously, these regulators have different or even opposite effects on the biological ability of ovarian cancer including proliferation, invasion, migration, and apoptosis under different mechanisms of action. This dynamic modification of RNA methylation reminds us of the importance of maintaining proper balance and avoiding excessive dysregulation of one regulator.

### Role of RNA methylation modifications in cancer stem cells, metastasis, tumour microenvironment, and immune infiltration

**Figure 3.**
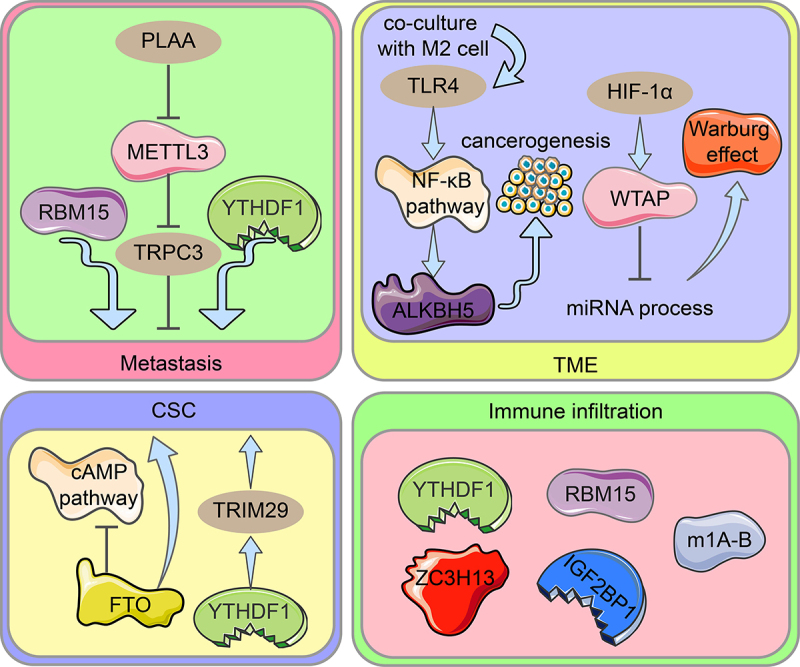
Diagram illustrating the role of RNA modification regulators in ovarian cancer spread, OCSC pathogenesis, TME, and immune infiltration. Metastasis: Through METTL3-mediated m6A alteration of TRPC3 mRNA, PLAA inhibits ovarian cancer metastasis. RBM15 encouraged metastasis of OC, while YTHDF1 inhibited it. CSC: In ovarian cancers and CSC, FTO expression is reduced, and the cAMP pathway which is important in stemness and tumour initiation is controlled. The cisplatin-resistant ovarian cancer cells’ stem cell-like phenotype is made possible by TRIM29 overexpression caused by m6A-YTHDF1. TME: TLR4 upregulates ALKBH5 expression in ovarian cancer cells co-cultured with M2 macrophages via activating the NF-B pathway, eventually driving ovarian carcinogenesis in the TME. Upregulation of WTAP expression by HIF-1α intercedes with miRNA processing, accelerates the Warburg impact. Immune infiltration: In ovarian cancer, YTHDF1, RBM15, ZC3H13, IGF2BP2, and the m1A-B cluster cloud mediate various degrees of immune infiltration.

Using various biological investigations, 21 m6A regulators were divided into two groups: those with low m6A scores and those with high m6A scores. Low m6A scores were associated with immune infiltration and responses to immune detection site inhibitors (ICIs) [91]. RNA modification patterns in ovarian cancer characterized by high immune infiltration and immune activation showed higher RNW scores, which were able to predict patient response to immunotherapy [74]. It has been suggested that m6A modification is linked to cellular immune infiltration and immune gene labelling. The m6A regulators RBM15, ZC3H13, YTHDF1, and IGF2BP1 may cause ovarian cancer immune infiltration [31]. In another study, phospholipase A2 activating protein (PLAA) degraded METTL3 via the ubiquitination pathway, which resulted in the destabilization of the m6A-modified mRNA oncogene TRPC3 and prevented the metastasis of ovarian cancer cells [92]. FTO is expressed at low levels in ovarian cancer stem cells (OCSC), and its overexpression inhibits OCSC proliferation, self-renewal and spherogenesis, and blocks cAMP signalling dependent on m6A demethylation [93]. TRIM29 expression levels correlate with prognosis in OC and enhance CSC-like features in cisplatin-resistant OC cells; YTHDF1 promotes TRIM29 translation in an m6A-dependent manner which contributes to the stem cell-like phenotype in cisplatin-resistant ovarian cancer [94]. Ovarian cancer cells cocultured with M2 macrophages showed the increased expression of the molecular toleration-like receptor (TLR4) in the tumour microenvironment (TME), which upregulated ALKBH5 by activating the NF-κB pathway, and eventually promoted ovarian carcinogenesis in the mock TME [82]. HIF-1α upregulation of WTAP expression interferes with miRNA processing and accelerates the Warburg effect (glucose metabolism by regulating glycolytic enzymes) in ovarian cancer in an m6A-dependent manner [86]. In addition, one study found that RBM15 upregulation and YTHDC1 downregulation were associated with OC cell metastasis [31]. In a retrospective study, GSEA found that high WTAP expression was enriched in MYC targets and cell cycle regulation-related pathways [67]. Different m1A modification patterns had various immune infiltration profiles, with m1A cluster B having the most abundant cellular immune infiltration and the greatest sensitivity to immunotherapy [80] (Figure 3). According to other studies, m6A methylation promotes immunosuppressive TME properties and supports tumour proliferation through pathways involving hypoxia, metabolic dysregulation, immune cells, and stem cell function. HIF affects tumour cells through m6A methylation modifications under hypoxic conditions. Tumour cells under hypoxia conditions promote the CSC phenotype and contribute to immunosuppressive TME formation. m6A-mediated metabolic dysregulation produces an acidic environment that further supports tumour growth and exacerbates tumour hypoxia. In addition, m6A methylation directly regulates immune cells and promotes the progressive establishment of an immunosuppressive TME.

## RNA methylation modifications as potential drug targets in ovarian cancer

Various studies have shown that RNA methylation is linked to tumour growth and may be used as a prognostic indicator for various cancers. FTO inhibitors have been most abundantly studied. For instance, Rhein was the first identified competitive inhibitor for FTO that can increase the cellular m^6^A on mRNA. It reversibly binds to the FTO or ALKB to form a complex and prevent the recognition of m6A substrates inside cells [95]. MA, a nonsteroidal anti-inflammatory drug, inhibits FTO demethylation of an m6A-containing ssDNA or ssRNA by inhibiting FTO rather than ALKBH5 [96]. Two FTO inhibitors discovered by using structure-based rational design, FB23 and FB23–2, can directly bind to FTO protein and selectively inhibit the m^6^A demethylase activity of FTO [97]. Similarly, two FTO inhibitors developed after screening, structural optimization and bioassays, 18077 and 18,097, selectively inhibited FTO demethylase activity and showed anticancer activity [98]. *R*-2-hydroxyglutaric acid (*R*-2HG) targets binding to FTO to inhibit demethylase activity and anti-tumour effects of 2HG in inhibiting the proliferation/survival of FTO-high cancer cells by targeting FTO/m6A/MYC/CEBPA signalling [99]. The nucleotide mimic with the tethered 2OG-binding component exhibited good potential in inhibiting FTO activity [100]. In addition, small molecule activators of METTL3 and inhibitors of METTL3, ALKBH5, and IGF2BP1 have been validated. tANK-binding kinase 1 (TBK1) interacts with METTL3 at serine 67 and phosphorylates METTL3 to promote its translational function [101]. S-adenosyl homocysteine (SAH) is an S-adenosyl methionine (SAM) dependent methyltransferase inhibitor. Eltrombopag noncompetitively binds to the metastable binding site on the METTL3-METTL14 complex to inhibit its m6A modification activity [102]. ALKH5 (ALK-04), a specific inhibitor of ALKBH5, enhances immunotherapeutic efficacy [103]. The small molecule compound ‘7773’ can directly bind to the RRM12 and KH34 structural domains on IGF2BP1 to affect the binding of other RNA targets [104]. Thus, various regulators of RNA methylation may serve as biomarkers for ovarian cancer diagnosis and potential drug targets for individualized therapy. Although inhibitors or activators of these RNA methylation regulators are expected to improve clinical outcomes for patients with tumours, insufficient experimental data and the lack of clinical trials will make it difficult for these therapeutic strategies to work.

**Figure 4.**
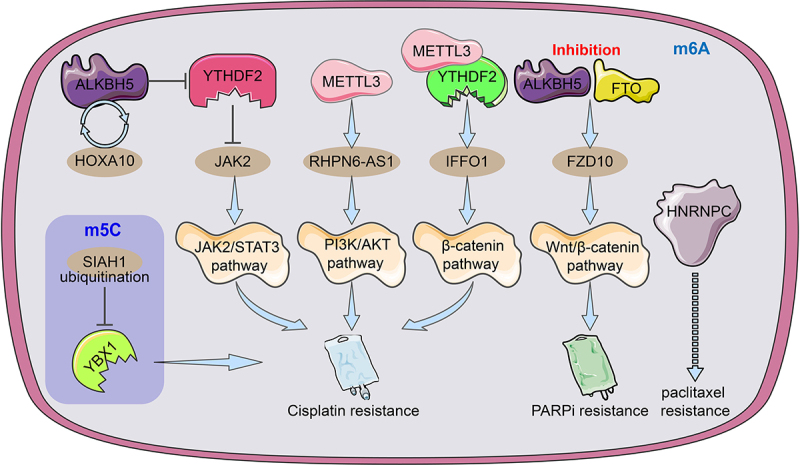
An illustration of the regulators of RNA alteration responsible for ovarian cancer medication resistance. The m6A regulator HNRNPC may be related to and be able to foretell OC resistance to paclitaxel. By facilitating JAK2 m6A demethylation, the ALKBH5-HOXA10 loop promotes EOC resistance to cisplatin by activating the JAK2/STAT3 signalling pathway simultaneously. METTL3-mediated m6A modification of RHPN1-AS1 accelerates cisplatin resistance by activating PI3K/AKT pathway. The METTL3/YTHDF2 axis regulated the mRNA stability of IFFO1 in an m6A-dependent manner and enhances cisplatin resistance. The ubiquitination of YBX-1 caused the target m5C-modified mRnas to become unstable, which made EOC cells more sensitive to the chemotherapy drug cisplatin. To increase FZD10 mRNA m6A modification, activate the Wnt/-catenin pathway, and improve PARPi resistance, it was necessary to downregulate the m6A demethylases FTO and ALKBH5.

In EOC, the HOXA10-ALKBH5 positive feedback loop promotes cellular cisplatin resistance *in vivo* and *in vitro*. ALKBH5 removes the m6A modification of JAK2 mRNA and inhibits YTHDF2-mediated JAK2 degradation to maintain JAK2 mRNA expression, which further activates the JAK2/STAT3 signalling pathway to promote EOC growth and cisplatin drug resistance [105]. m6A-modified FZD10 mRNA was upregulated in PARPi-resistant cells and knockdown of FZD10 inhibited Wnt/β-catenin signalling to sensitize drug-resistant cells to PARPi drugs. In addition, inhibition of FTO and ALKBH5 reduced m6A modification which contributed to the upregulation of m6A-FZD10 mRNA [106]. METTL3-mediated m6A modification of lncRNA RHPN6-AS1 enhances cisplatin resistance in ovarian cancer through activation of the PI3K/AKT pathway [84]. The METTL3/YTHDF2 axis promotes degradation of tumour suppressor IFFO1 mRNA in an m6A-dependent manner to promote nucleation of β-catenin and enhance cisplatin resistance in ovarian cancer [107]. HNPNPC was considered a predictor of paclitaxel resistance based on the 6-month relapse-free survival (RFS) of ovarian cancer chemotherapy [31]. In addition, the ubiquitination of the m5C ‘reader’ YBX1 resulted in the destabilization of its modified mRNA and reduced EOC resistance to cisplatin [108] (Figure 4). MiR-152 and miR-185 jointly promote cisplatin sensitivity in ovarian cancer cells by directly targeting DNMT1 [109]. Negative feedback between miR-143 and DNMT3A regulates cisplatin resistance in ovarian cancer [110]. M7G methylation has also been shown to be involved in tumour chemoresistance. For instance, low METTL1 expression alleviated nasopharyngeal carcinoma resistance to cisplatin and doxorubicin and cervical cancer resistance to 5-fluorouracil, and WRD4 enhanced sorafenib resistance in hepatocellular carcinoma by promoting the EMT process [111–113]. In short, these modulators of RNA methylation as oncogenes (cancer suppressor genes) or targeted proteins to regulate chemotherapy resistance in ovarian cancer, which are expected to become new biomarkers and drug targets for cancer therapy.

## Conclusion and future directions

Overall, the following variables determine how RNA modification of target genes affects the development of ovarian cancer: 1) whether the regulator is an oncogene-promoting or oncogene-suppressing gene; 2) the abnormal level of RNA modification in cancer; and 3) the regulatory mechanism of the target mRNA modification. On account of various methylation modification regulators have different biological properties, some small molecule inhibitors or activators targeting RMPs have emerged that can be involved in regulating the level of RNA modification. It is predicted that RNA methylation might serve as a prospective molecular marker for early diagnosis and individualized treatment of cancer. However, RNA modifications show differences in different cancers or cell lines. Although m6A modifications have been extensively studied in various other cancers, research on RNA methylation in ovarian cancer is still in the preliminary stage.

Epigenetic transcriptional regulation mainly involves DNA methylation, histone modifications and chromatin remodelling. Dysregulation of DNA methylation and histone deacetylation or methylation are the most intensively studied epigenetic changes in cancer and their dysregulation contributes to cancer progression. DNMTs are the enzymes responsible for DNA methylation, of which DNMT1, DNMT3A and DNMT3B have methyltransferase activity [114]. Currently, two DNA methylation reagents based on nucleoside analogues are azacytidine (AZA, 5-azacytidine) and decitabine (DAC, 5-AZA-dC). These two DNA methylation reagents have been approved by the FDA for myelodysplastic syndromes (MDS) and chronic myelomonocytic leukaemial. Several other novel DNMT inhibitors (DNMTi) are also emerging in clinical therapy. Guadecitabine (SGI-110, Astex), hydrazine, and 5-fluoro-20 -deoxycytidine (FdCyd) are all being used in clinical trials [115–117]. Some studies have reported that azacytidine can affect RNA metabolism by interfering with the synthesis, stability and function of rRNA, mRNA and tRNA. There are several clinical trials using DNMTi in the treatment of OC patients.

Histone lysine methyltransferases (KMTs) label histone lysines and create substrates for histone demethylases. KMTs are similar to DNMTs and catalyse methylation labelling with SUV39H1, DOT1L, and EZH2, a member of the multicomponent protein family. EPZ-5676, a DOT1L inhibitor, targets S-adenosine homocysteine analogues and has been used in clinical trials in AML. Two inhibitors of EZH2, EPZ-6438 and CPI-1205, have both been applied in clinical trials for different tumours. Lysine demethylases (KDM) contain the KDM1 subfamily of lysine-specific demethylases (LSD) and the KDM2–7 subfamily containing the Jumonji C (JmjC) structural domain [118,119]. Studies showing that monoamine oxidase (MAO) inhibitors inhibit LSD1 have been widely used to support clinical trials [120]. The LSD1 inhibitor GSK2879552 inhibits small cell lung cancer (SCLC) and AML cell proliferation and predicts the sensitivity of SCLC-derived xenografts to small molecule inhibitors [121]. In addition, the non-competitive inhibitor 2d (verlindamycin) and the competitive inhibitor oligomeric amine PG-11144 showed antitumour effects [122–124]. Impressively, the combined pairing of these key enzyme inhibitors resulted in better anticancer effects. In conclusion, dysregulation of DNA methylation and posttranslational histone modification processes is an important target for therapeutic intervention in cancer.

RNA modifications interact inextricably with epigenetic modifications. DNA methylation directly controls the genetic expression of m6A regulators. In pancreatic duct epithelial cells, cigarette smoke condensate causes hypomethylation of the METTL3 promoter, induces METTL3 expression and promotes PC development and progression [125]. In addition, RNA methylation and DNA methylation share the same methyl donors and epigenetic marks. RNA methylation selectively acts on transcripts encoding epigenetic modifiers of histone modifications to regulate histone modifications. YTHDC1 recruits the H3K9me2 demethylase KDM3B to m6A-associated chromatin regions to regulate local H3K9me2 levels and gene expression [126]. Histone modifications mediate RNA m6A methylation at the posttranscriptional and transcriptional levels. Suppression of METTL14 is triggered by H3K2me4 loss induced by methyltransferase SETD3 knockdown or demethylase KDM36A overexpression, leading to overall m6A demethylation [127].

Unlike DNA methylation and histone modifications, RNA methyltransferases and their inhibitors have not been studied thoroughly enough. Some inhibitors of FTO and several inhibitors of METTL3 and ALKBH5 have been found to exert regulatory effects on RNA methylation modifications. The lack of *in vivo* and *in vitro* cellular experiments and clinical trials in cancer implies the great potential of RNA methylation in cancer therapy. Perhaps, the development of effective RNA methylation inhibitors or demethylating agents may provide prospective therapeutic strategies for OC and recurrent drug resistance.

## Data Availability

Not available.
